# Luminescence mechanism in hydrogenated silicon quantum dots with a single oxygen ligand†

**DOI:** 10.1039/d0na00986e

**Published:** 2021-02-26

**Authors:** Hong Shen, Zhiyuan Yu, Jinjin Wang, Ming Lu, Chong Qiao, Wan-Sheng Su, Yuxiang Zheng, Rongjun Zhang, Yu Jia, Liangyao Chen, Caizhuang Wang, Kaiming Ho, Songyou Wang

**Affiliations:** Shanghai Ultra-Precision Optical Manufacturing Engineering Center, Department of Optical Science and Engineering, Fudan University Shanghai 200433 China songyouwang@fudan.edu.cn; National Taiwan Science Education Center Taipei 11165 Taiwan wssu@mail.ntsec.gov.tw; Department of Electro-Optical Engineering, National Taipei University of Technology Taipei 10608 Taiwan; Key Laboratory for Special Functional Materials of Ministry of Education, Collaborative Innovation Center of Nano Functional Materials and Applications, School of Materials Science and Engineering, Henan University Kaifeng Henan 475001 China; Ames Laboratory, U. S. Department of Energy and Department of Physics and Astronomy, Iowa State University Ames Iowa 50011 USA; Key Laboratory for Information Science of Electromagnetic Waves (MoE) Shanghai 200433 China

## Abstract

Though photoluminescence (PL) of Si quantum dots (QDs) has been known for decades and both theoretical and experimental studies have been extensive, their luminescence mechanism has not been elaborated. Several models have been proposed to explain the mechanism. A deep insight into the origin of light emissions in Si QDs is necessary. This work calculated the ground- and excited state properties of hydrogenated Si QDs with various diameters, including full hydrogen passivation, single Si=O ligands, single epoxide and coexisting Si=O and epoxide structures in order to investigate the dominant contribution states for luminescence. The results revealed that even a single oxygen atom in hydrogenated Si QDs can dramatically change their electronic and optical properties. Intriguingly, we found that a size-independent emission, the strongest among all possible emissions, was induced by the single Si=O passivated Si-QDs. In non-oxidized Si-QDs exhibiting a core-related size-tunable emission, the luminescence properties can be modulated by the ligands of Si QDs, and a very small number of oxygen ligands can strongly influence the luminescence of nanocrystalline silicon. Our findings deepen the understanding of the PL mechanism of Si QDs and can further promote the development of silicon-based optoelectronic devices.

## Abstract 2

Upon excitation, electrons are strongly localized near the Si=O site, which induces a faster radiative transition.

## Graphical abstract

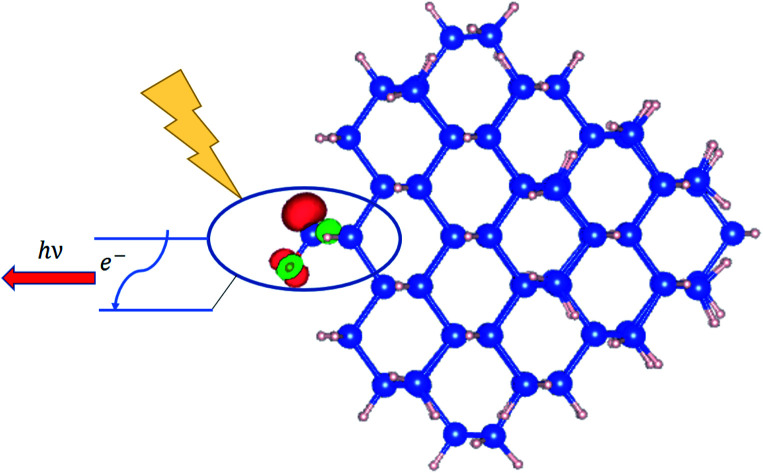

## Introduction

Over the past few decades, silicon has been widely used in various devices in the microelectronic and photovoltaic industries due to its abundancy and non-toxicity. However, its indirect band nature has limited its application in the areas of photochemistry and photophysics, such as in light-emitting devices. Unlike bulk silicon, silicon at the nanoscale has been considered as a promising light source candidate since the discovery of porous silicon luminescence by Canham *et al.*^1^ This area has been developing rapidly over the years. Research on Si-light emitting diodes (LEDs) has achieved a high quantum yield as well as a narrow bandwidth.^2^ Recently, the world's first all silicon laser was developed using silicon nanocrystals with high optical gains.^3,4^ Although the silicon-based laser light source has made important progress, there is still a lot of work to be done before it can be applied.

Much effort has been made from both experimental and theoretical perspectives to pursue the origin of luminescence in Si-QDs. On the experimental side, complex reasons were found to affect the luminescence of silicon quantum dots (QDs), such as the size of Si-QDs,^5–10^ passivation of dangling bonds,^11–17^ surface tension^18,19^ and temperature.^20,21^ On the theoretical side, Proot *et al.*^5^ demonstrated that the quantum confinement effect dramatically increases the band gaps of silicon QDs due to strongly confined electrons in all three directions. Though the momentum *k*-conservation is relaxed, the transition still shows an indirect nature and the observed photoluminescence (PL) lifetime is found to be at the scale of tens or hundreds of microseconds.^22–25^ In contrast, the PL lifetime of direct QDs only reaches a few nanoseconds, according to previous experimental results.^22^ Despite much effort, the mechanism behind luminescence is still unclear, especially the role played by the passivation ligand, and this is of great interest and practical importance.

The quantum confinement effect provides a clear physical picture why small QDs may be emitted. In experiments, however, it was found that the band gap of Si-QDs violated the quantum confinement effect, especially for ultra-small QDs.^25,26^ It turns out that oxygen plays an important role in both Si-QDs and all-silicon lasers.^4^ During fabrication of devices, it is nearly inevitable to have a some amount of oxygen becoming attached to the surface of Si-QDs, especially for those embedded in silica. Wolkin *et al.*^27^ demonstrated that the gaps between the highest occupied molecular orbital (HOMO) and lowest unoccupied molecular orbital (LUMO) of Si-QDs of different sizes are nearly size-independent in the presence of the Si=O ligand on the surface of hydrogenated Si-QDs by means of a tight-binding model, inducing localized exciton recombination at the band edge. In their work, the only ligand considered was Si=O, though there are many ways to oxidize a Si-QD even for a single atom ligand, such as the Si=O covalent bond, a single epoxide, and a co-existing Si=O and epoxide structure. Some questions remain: How do these different combinations of oxygen and hydrogenated Si-QDs affect their luminescence performance? To what extent can single oxygen atoms affect the optical properties of Si-QDs?

In order to answer the questions, in this work, we have explored both the ground state and excited state properties of Si-QDs with DFT and TD-DFT simulations, respectively. We investigated the excitation properties and emission from the excited states (S_1_ or higher) to the ground state (S_0_) of Si-QDs with different diameters and different passivation conditions. Nearly size-independent emission energy was found, but only in Si-QDs containing single Si=O passivation, such that these Si-QDs which exhibited the strongest emission intensity among all conditions. Unlike findings from previous research which showed that Si-QDs have an emission lifetime of several hundreds of μs, a more rapid radiative transition was identified in these Si-QDs. These computational results provide a better understanding of PL in Si-QDs.

## Calculation method

The simulations were carried out using a quantum chemistry package ORCA based on the density functional theory (DFT) and time-dependent density functional theory (TD-DFT).^28^ The all-electron Gaussian basis of balanced polarized triple-zeta basis (def-TZVP)^29^ and hybrid exchange-correlation functional put forward by Becke, Lee, Yang and Parr (B3LYP/G)^30,31^ were used. The Si-QDs investigated in this study were cut from the diamond-phase bulk silicon with a diameter of 1.1, 1.3, 1.7 and 2.0 nm, containing 35, 66, 124 and 220 silicon atoms, respectively. In this work, the Si-QDs chosen were not completely spherical. For each atom on the surface, there were either one or two dangling bonds to be passivated depending on position. The atoms on the (1 1 0) facet had only one dangling bond while those at the intersection of two facets had two. Surface dangling bonds were passivated either by H or O atoms. Then all QDs were relaxed until force was less than 3 × 10^−4^ Eh Bohr^−1^ to obtain the stable structure. For excited state properties, linear-response TD-DFT^32^ calculations were carried out. Specifically, emission from the S_1_ state was given by standard TD-DFT, while absorption spectra were calculated by a simplified TD-DFT (sTD-DFT) which gives a reasonable spectrum, as invented by Grimme.^33^ The emission spectrum is obtained by smearing the oscillator strength by a Gaussian function. Oscillator strength is used to quantify the probability of whether a certain transition can take place. The oscillator strength *f*_12_of a transition between states |1〉 and |2〉 is defined by1
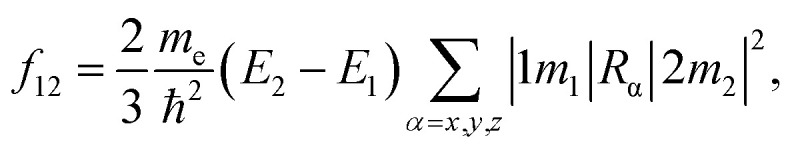
where *m*_e_ is the mass of an electron and ℏ is the reduced Plank constant. The quantum states |1*m*_1_〉 and |2*m*_2_〉 were used to denote two states involved in the transition. To accelerate the simulation, the resolution of identity approximation (RI) implemented in ORCA was applied.^34^

## Results and discussion

**Fig. 1:**
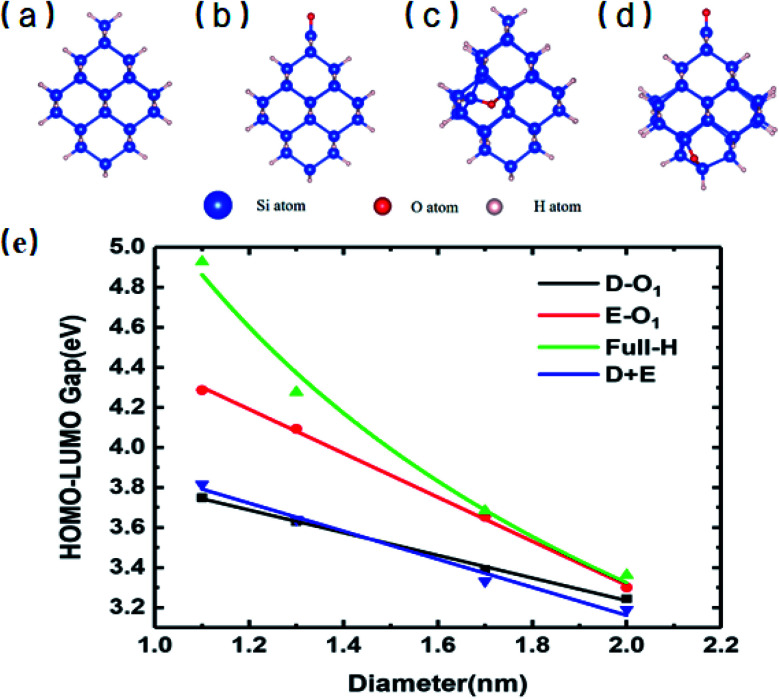
Structures of Si-QDs with a diameter of 1.1 nm and HOMO–LUMO gaps of QDs with different diameters and passivation configurations: (a) 1.1-H_36_, (b) 1.1-D-O_1_, (c) 1.1-E-O_1_, (d) 1.1-D + E, and (e) HOMO–LUMO gaps. The symbols represent the calculated HOMO–LUMO gap of Si-QDs of specific sizes with corresponding passivation conditions. The curves were fitted by an exponential function or linear function.

Fig. 1 shows the specific structures of Si-QDs with a diameter of 1.1 nm containing different passivation configurations (a–d) and their HOMO–LUMO gaps of Si-QDs with different diameters (e). For simplicity, notations are used to describe the structures of QDs. The structures saturated only by hydrogen atoms are denoted as *m*-H_*n*_ (*m* is the diameter of QDs, and *n* is the number of hydrogen atoms), such that the QDs shown in Fig. 1(a) are named as 1.1-H_36_. Similarly, larger QDs can be named as 1.3-H_64_, 1.7-H_96_ and 2.0-H_144_, as shown in Fig. S1 in the ESI†. From Fig. S1,† it can be seen that the structure of 1.3-H_64_ is slightly different from other non-oxidized Si-QDs, especially the uppermost part, which may lower the symmetry of QD leading to a longer emission lifetime. The cases with a double bonded oxygen atom and single epoxide ring can be written as *m*-D-O_1_ and *m*-E-O_1_, respectively. Likewise, the QDs having coexisting Si=O and epoxide were denoted as *m*-D + E. All QDs illustrated in the SM are denoted in a similar way. There may be several different structures for the same chemical formula, especially for epoxide structures and large QDs, but only the most stable ones of each chemical formula and their results are shown in the main work. The corresponding sketches of their structures are shown in the ESI (Fig. S1†). Other structures are taken into consideration and their corresponding results are collected in the SM.

Fig. 1(e) shows the gaps between the HOMO and LUMO of different sizes and passivation conditions. It shows an overall trend where the HOMO–LUMO gaps increase with decreasing QD sizes for Si-QDs passivated by hydrogen, predicted by the quantum confinement (QC) effect, which can be depicted by a decreasing exponential curve. Similar qualitative results have been reported,^5,26,35–38^ but the results differ slightly with the calculation method. For example, the standard DFT with LDA functional^36^ and the empirical pseudopotential method (EMP)^5,26^ gave smaller gaps compared with DFT combined with hybrid functional B3LYP.^35^ It is known that hybrid functionals like B3LYP give a more accurate picture of band gaps than the LDA or GGA functional do. In this manuscript, the B3LYP functional was utilized throughout all calculations. When Si-QDs were oxidized, however, a notable decrease in H–L gaps was observed at the same size, and variations with respect to size were linear. Puzder *et al.*^36^ also showed that, upon oxidization by Si=O, the gap of 1.1 nm abruptly changed from 3.4 eV to 2.2 eV, and the size dependence of gaps for oxidized Si-QDs showed a linearly dependent gap change. Our results in Fig. 1(e) are qualitatively consistent with ref. 36, and it can be noted that the size dependence of gaps of oxidized Si-QDs is linear for both Si=O and Si–O–Si, and the slope of Si=O is much smaller than that of Si–O–Si. Though the absolute value of gaps is different, the slope of our result is similar compared to ref. 36, with the numerical difference due to different exchange–correlation functionals chosen in calculation. Among all Si-QDs, the non-oxidized ones exhibit the most apparent gap variation with respect to size. For the oxidized ones, the variation of H–L gaps with respect to size was quite small, which may be attributed to deep impurity levels induced by oxygen. These results are consistent with the literature as reported by previous calculations.^27^

**Fig. 2:**
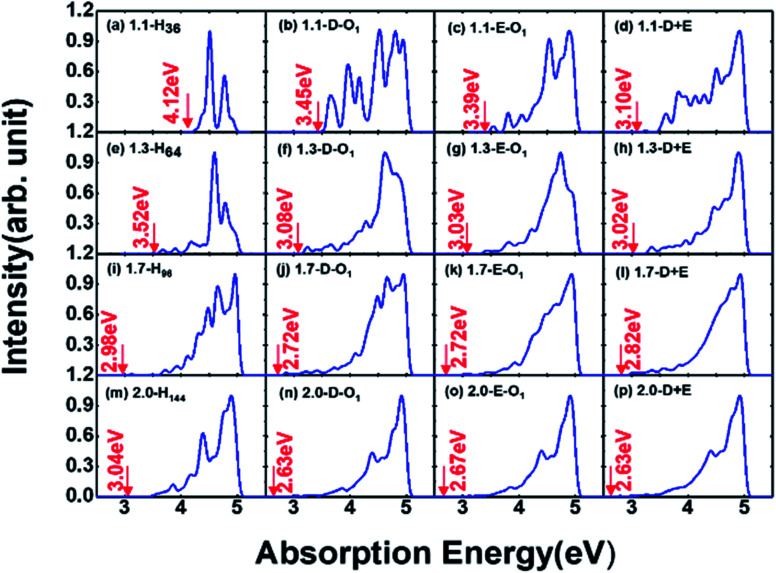
Absorption spectrum of QDs of different diameters and passivation conditions. Each column represents different diameters. (a–c) Absorption spectra of 1.1 nm QDs of non-oxidized, single Si=O passivated, and single epoxide structures, respectively. Absorption spectra of (d–f) 1.3 nm QDs, (g–i) 1.7 nm QDs, and (j–l) 2.0 nm QDs.

Absorption properties are important for light-emitting materials, especially for light-driven luminescence materials like Si-QDs. Theoretically, the DFT calculation is an effective tool to study the ground state properties of Si-QDs, but it fails to describe the excitation properties. To this end, TD-DFT simulations are performed to study the absorption and emission spectra from the first excited singlet state (S_1_ state). Fig. 2 shows the absorption spectrum for different configurations and different sizes.

Different rows in Fig. 2 represent different sizes of Si-QDs from 1.1 to 2.0 nm, while columns represent full-H, D-O_1_, E-O_1_ and D + E, respectively. The absorption edge of each Si-QD is marked by red arrows. The first column of Fig. 2 shows that the absorption edge decreases as the diameter of Si-QDs increases for hydrogen passivated Si-QDs, which is consistent with the prediction of the quantum confinement effect. But when Si-QDs were oxidized, regardless of the way oxygen attaches to the surface, even by a single oxygen atom, the absorption edge will red shift. As seen from the three columns of oxidized Si-QDs, the absorption edge is also size-dependent, but quantitatively less compared to hydrogen passivated ones. The presence of O-passivation red shifts the absorption edge of Si-QDs, which is consistent with results shown in Fig. 1.

**Fig. 3:**
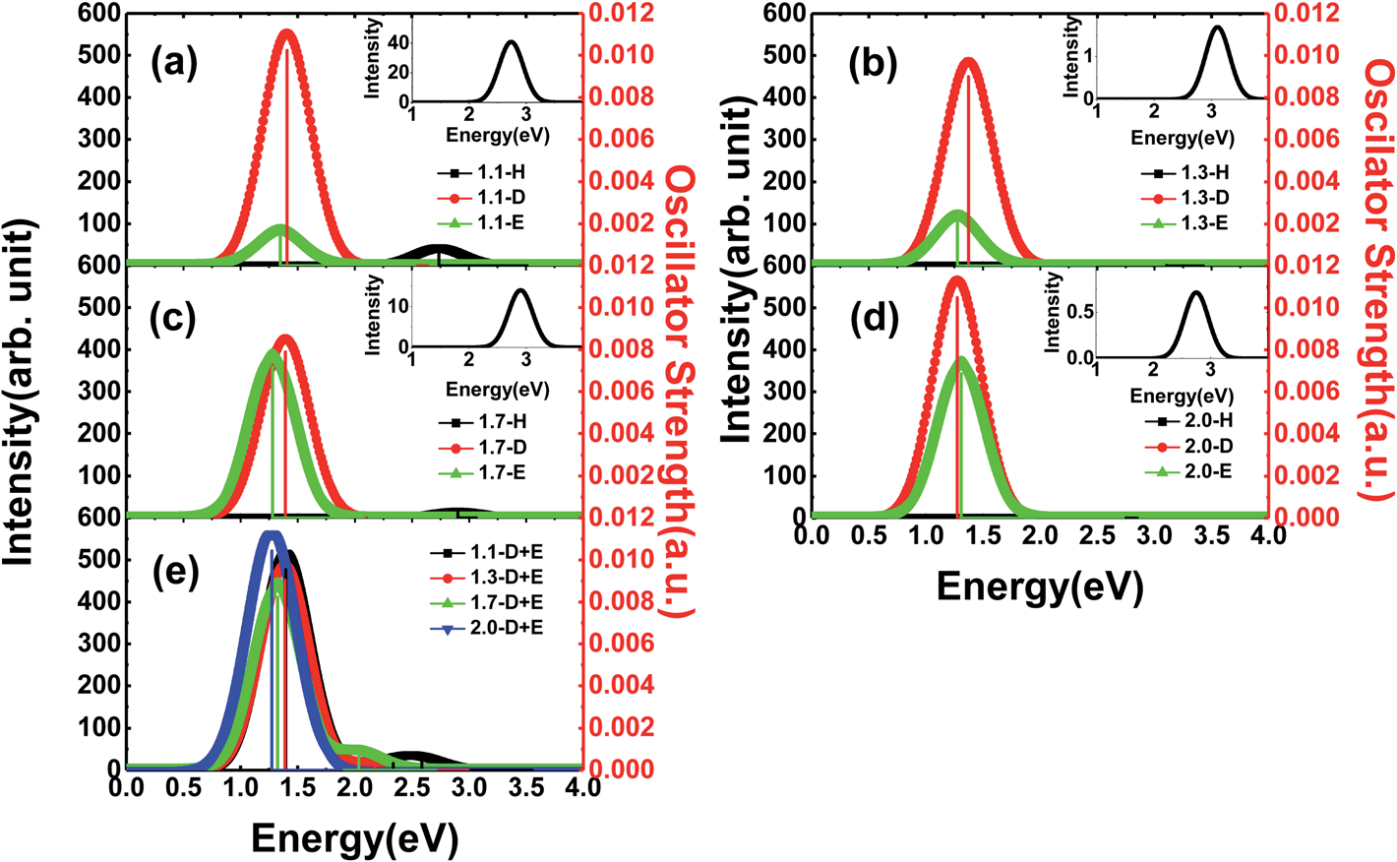
Emission spectrum and oscillator strength of Si-QDs from S_1_ to S_0_ states. Vertical line indicates the oscillator strength. Insets are spectra of hydrogen passivated Si-QDs: (a) 1.1 nm Si-QDs, (b) 1.3 nm Si-QDs, (c) 1.7 nm Si-QDs, (d) 2.0 nm Si-QDs, and (e) coexisting Si=O and epoxide passivation structures for different sizes from 1.1 to 2.0 nm.

In general, Si-QDs can be regarded as a species that obeys Kasha's rule, which states that the fluorescence emission spectrum is generally independent of the excitation wavelength^39,40^ This means only radiative recombination between the S_1_ and S_0_ states contributes to fluorescence. Hence, the emission from *S*_1_ to *S*_0_ is considered in our investigation, except for *m*-D + E Si-QDs, where higher state emission is considered to distinguish the contribution of the Si=O ligand and epoxide structure. As illustrated in Fig. 3, the vertical line indicates the oscillator strength, where it is clear that different Si-QD configurations have notable differences in *S*_1_ emission. Fig. 3(a–d) represent the emission spectra of different Si-QDs from 1.1 nm to 2.0 nm, respectively. Black, red and green lines are used to depict different kinds of passivation conditions: fully hydrogen, single Si=O ligand and epoxide structure, respectively. Fig. 3(e) shows the emission spectrum of QDs with coexisting Si=O and epoxide structure. It is clear that the QDs with a single Si=O ligand exhibit the strongest fluorescence intensities at all sizes and these intensities do not change much with respect to size. In comparison, the epoxide structure has similar emission energies, but the emission intensity grows with size. The fully hydrogen passivated QDs show the highest emission energy (as in the inset of Fig. 3), suggesting that the oxidation of QDs lowers the emission energy. The oscillator strength of these non-oxidized QDs is almost at the scale of 1 × 10^−5^, specifically 0.74 × 10^−5^, 3.12 × 10^−5^, 0.26 × 10^−5^ and 1.32 × 10^−5^ from 1.1-H_36_ to 2.0-H_144_, respectively. The corresponding emission energies are 2.69, 3.11, 2.90 and 2.75 eV.

One interesting feature in Fig. 3 is that the emission energy of all structures containing only one Si=O ligand exhibited nearly identical emission energy even for the largest QD containing more than 300 atoms. The deviation of emission energy with respect to size of hydrogen passivated Si-QDs can be as large as 0.42 eV, much greater than that of double bonded QDs (0.05 eV), indicating that the emission energies are less affected by size for small Si-QDs. These results theoretically confirm the explanations put forward by Wolkin.^27^ This size-independent phenomenon means that radiative transitions which occurred in these QDs containing Si=O bonds are between localized electronic states. In addition, from the emission intensity shown in Fig. 3, we can speculate that these localized transitions dominate in the luminescence of QDs. The results show that the Si=O ligand plays a decisive role in the luminescence of small size silicon QDs. Further, fluorescence properties of QDs with coexisting Si=O and epoxide are shown in Fig. 3(e), where multiple peaks can be seen in the spectrum. The strongest peaks are located near 1.4 eV, which is close to the emission energy of double bonded structures. Only minor variation with respect to size was observed. The smaller peaks move to a lower energy regime for larger QDs and the intensities are negligible compared to the main peak. According to excited state analysis, the emission from S_1_ was mainly contributed by the HOMO and LUMO while higher state emission considered in this work was contributed by HOMO − 1 (the molecular orbital one level lower than HOMO) and LUMO + 1 (molecular orbital one level higher than LUMO). It can be seen that the HOMO and LUMO are centered near the Si=O ligand while HOMO − 1 and LUMO + 1 are centered near the Si–O–Si ligand (Fig. S2†).

**Fig. 4:**
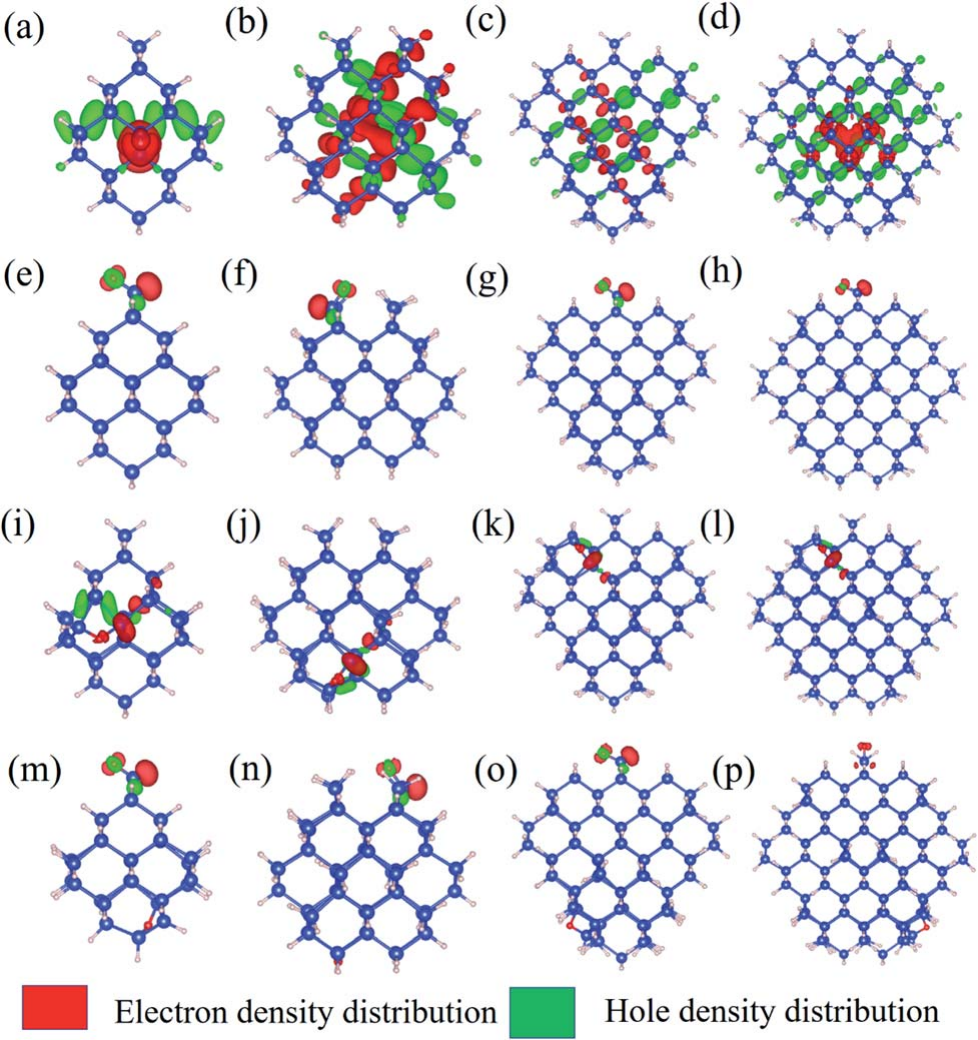
Electron–hole density distribution in Si-QDs: (a) 1.1-H_36_, (b) 1.3-H_64_, (c) 1.7-H_96_, (d) 2.0-H_220_, (e) 1.1-D-O1, (f) 1.3-D-O1, (g) 1.7-D-O1, (h) 2.0-D-O1, (i) 1.1-E-O1, (j) 1.3-E-O1, (k) 1.7-E-O1, (l) 2.0-E-O1, (m) 1.1-D + E, (n) 1.3-D + E, (o) 1.7-D + E, and (p) 2.0-D + E.

To further explore the feature of the emission spectrum, the electron–hole density distribution is presented in Fig. 4, where the red regions represent the distribution of electron density, while the green ones represent hole density distribution. The iso-values are chosen to be slightly different to provide a better exhibition of electron–hole distribution. The iso-values of all oxidized QDs are all chosen to be 0.005 (Fig. 4(e–p)), while 1.1-H_36_ has a slightly smaller iso-value of 0.001 (Fig. 4(a)) and larger non-oxidized QDs have an even smaller iso-value of 0.0005 (b–d). The first row of Fig. 4 shows the electron–hole distribution of non-oxidized Si-QDs of different diameters. It can be observed that the electron and hole densities are spread over the Si-QDs. On the contrary, the second row gives electron hole density distribution in Si-QDs of various sizes which contain a single Si=O ligand. Obviously, all electrons and holes participating in radiative recombination concentrate near the oxygen ligand. This feature does not change with respect to the Si-QD size. The electron and hole densities tend to distribute more evenly for larger QDs (Fig. 4(b–d)). From Fig. 4(e–h), these QDs exhibited Si=O induced radiative transitions which took place between regions near the O ligand. This strong localization of carriers regardless of size indicates that the Si=O ligand is most responsible for emission. Thus, it is not surprising to see a size-independent emission due to similar carrier behavior in Si-QDs of all sizes. In contrast, as seen from the first row of Fig. 4, the electron and hole density distributions are highly non-localized, which suggests that the emission character must be dependent on size, and accordingly, size-tunable emission exists in hydrogen passivated Si-QDs. Electron and hole density distribution sheds light on the origin of emission energy and intensity difference between non-oxidized and single oxygen oxidized Si-QDs. The localization of carriers in real space due to the presence of this O defect increases their uncertainty in momentum space, according to Heisenberg's uncertainty principle. Since momentum conservation is relaxed, the existence of O-defects lifts the oscillator strength of QDs because of the facilitation of the quasi-direct transition. Further, due to the similarity of the physical properties and chemical environment of the single Si=O ligand on the Si-QD surface, in addition to its dominating contribution to radiative recombination, the emission signatures must be closely related to the single oxygen ligand rather than the size of Si-QDs. This explains the size-independent feature of emission in Si-QDs with only one oxygen. In contrast, the fully hydrogen-passivated QDs show a less localized electron–hole density distribution, indicating that the transitions took place over the whole Si core region. Therefore, the transitions in these QDs exhibit a slow character and originate from the QC effect.

The third and fourth rows of Fig. 4 show the electron–hole distribution of the epoxide ligand and coexisting Si-QDs. Fig. 4(i–l) show that the epoxide structure could also confine electrons and holes but to a weaker extent than that of the Si=O ligand. This can be deduced by comparing the second and third rows of Fig. 4, in which electron hole distribution is more confined in QDs containing the Si=O ligand. This feature may be attributed to the physical difference between the Si=O bond and Si–O–Si bond. Fig. 4(m–p) depict the electron hole density distribution for coexisting Si-QDs. It shows similar patterns to Si=O ligand ones where the majority of carriers take part in radiative recombination induced by the Si=O ligand. Thus, even where the double bond and epoxide oxygen ligand coexist, the strongest emission intensity is contributed by the Si=O ligand. This means that Si=O has the most dominant effects on QDs, resulting in a stronger fluorescence intensity.

Apart from emission energy and intensity, lifetime is another vital parameter to evaluate a good emitter. According to Einstein coefficients, the lifetime of spontaneous emission is related to the energy difference between two states involved in the emission process as well as oscillator strength. The spontaneous emission factor can be written as2
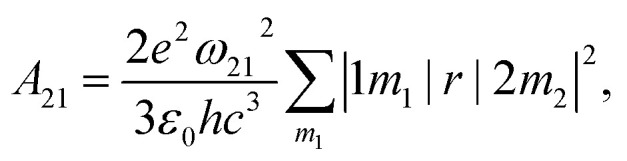
where the energy difference is encoded in coefficient 
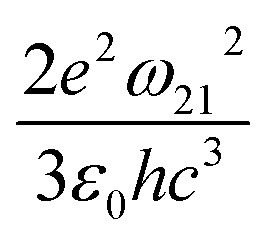
. The norm square is used to describe the dipole moment between the initial state |2*m*_2_〉 and final possible states |1*m*_1_〉, which is closely related to the oscillator strength. The summation over result *A*_21_ is the reciprocal of emission lifetime.^41^3
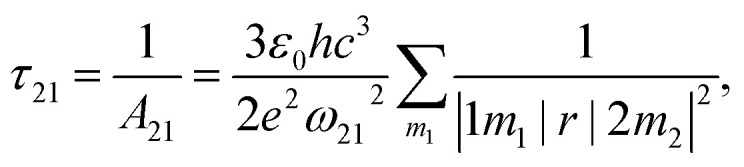


**Fig. 5:**
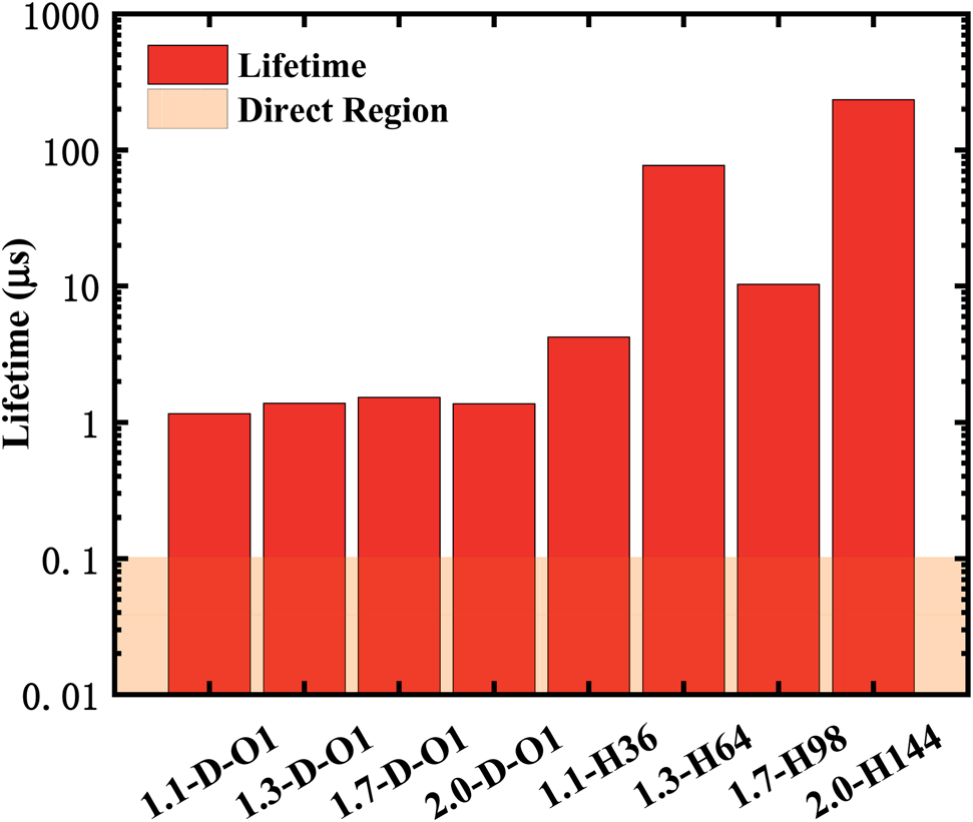
Lifetime of the emission of Si=O double bonded and hydrogen-passivated Si-QDs of different diameters.

According to eqn (2) and (3), the emission lifetime of Si=O double bonded and hydrogen-passivated Si-QDs of different diameters can be calculated and the results are given in Fig. 5, where it can be seen that hydrogen passivated Si-QDs have an emission lifetime of around 4 to 200 μs, exhibiting an indirect band gap feature,^22–25^ which varies with respect to size. It seems abnormal that 1.3-H_64_ has even lower oscillator strength as well as longer emission lifetime compared to larger hydrogen passivated Si-QDs. In contrast, when the Si-QDs are oxidized by even a single Si=O ligand, the lifetime dramatically decreases by nearly two orders of magnitude and is clearly size-independent. The radiative transition was mainly localized near the Si=O ligand as demonstrated in previous analysis of Fig. 3, and therefore the lifetime of these Si = QDs was decided by the character of Si=O. Thus, Si-QDs containing Si=O were nearly independent. On the contrary, emission properties of non-oxidized Si = QDs were highly size-dependent as shown in Fig. 3. In addition, the structure symmetry plays a role, which led to the non-monotonic behavior. More thorough exploration of this issue should be addressed in future work. In order to be a candidate for the laser material, the lifetime can be neither too long nor too short. On the one hand, a too long lifetime limits emitting efficiency; on the other hand, too fast radiative recombination restricts optical gain which is fatal in lasering. The pale orange panel in Fig. 5 indicates the lifetime of an ordinary direct band gap material.^42^ Thus, the oxidized Si-QD lifetime lies between the indirect and direct regions, and can be identified as a quasi-direct recombination.

## Conclusions

In this work, DFT and TD-DFT were implemented to explore the mechanism of fluorescence in Si QDs. Si-QDs of different surface conditions were considered, including hydrogen-passivated and oxidized with single oxygen ligands. The HOMO–LUMO gaps of Si-QDs showed a typical QC effect for hydrogen passivated ones while a nearly linear trend corresponding to size was observed for oxidized ones. Interesting features were observed in emission spectra. For hydrogen-passivated Si-QDs, they inherit the indirect recombination nature from bulk silicon, resulting in a constrained emission intensity and long recombination lifetime. The scenario was much different when a single oxygen atom was attached to the surface of hydrogenated Si-QDs. For those containing the single Si=O ligand, the emission energy was size-independent and exhibited strong emission intensity. In addition, the epoxide structure Si-QDs also showed relatively size-independent emission energy while the emission intensity was very small in smaller Si-QDs (1.1 and 1.3 nm) compared to those induced by the Si=O ligand. But the intensity dramatically increased when the sizes were slightly larger. When they coexisted with one QD, the emission was dominated by Si=O. Through the analysis of electron–hole density distribution, it can be concluded that the high emission intensity induced by Si=O can be attributed to the relaxation of momentum conservation in recombination due to the strong localization of carriers in real space. The emission lifetime of this kind of recombination lies between indirect and direct and can be identified as a quasi-direct recombination. Our results give a better understanding of the mechanism of luminescence in Si-QDs, which provides a theoretical foundation to help the development of all-silicon photoelectric devices.

## Conflicts of interest

There are no conflicts to declare.

## Supplementary Material

NA-003-D0NA00986E-s001
