# Supplementary material for: Luminescence mechanism in hydrogenated silicon quantum dots with a single oxygen ligand
Source: Nanoscale Adv. 2021 Feb 26;3(8):2245–51. doi: 10.1039/d0na00986e (PMC9417146; doi:10.1039/d0na00986e)
Supplement: NA-003-D0NA00986E-s001 [file NA-003-D0NA00986E-s001.pdf]

Electronic Supplementary Material (ESI) for Nanoscale Advances.
This journal is © The Royal Society of Chemistry 2021

Supporting Information

Luminescence Mechanism in hydrogenated Silicon Quantum Dots with Single Oxygen Ligand

Hong Shen[a], Zhiyuan Yu[a], Jinjin Wang[a], Ming Lu[a], Chong Qiao[a], Wan-Sheng Su[b,], Yuxiang Zheng[a], Rongjun Zhang[a], Yu Jia[c], Liangyao Chen[a], Caizhuang Wang[d], Kaiming Ho[d], Songyou Wang[a,e] **

[a.] *Shanghai Ultra-Precision Optical Manufacturing Engineering Center, Department of Optical Science and Engineering, Fudan University, Shanghai 200433, China.*

[b.] *National Taiwan Science Education Center, Taipei 11165, Taiwan and Department of Electro-Optical Engineering, National Taipei University of Technology, Taipei 10608, Taiwan.*

[c.] *Key Laboratory for Special Functional Materials of Ministry of Education, Collaborative Innovation Center of Nano Functional Materials and Applications, and School of Materials Science and Engineering, Henan University, Kaifeng, Henan 475001, China.*

[d.] *Ames Laboratory, U. S. Department of Energy and Department of Physics and Astronomy, Iowa State University, Ames, Iowa 50011, USA*

[e.] *Key Laboratory for Information Science of Electromagnetic Waves (MoE), Shanghai 200433, China*

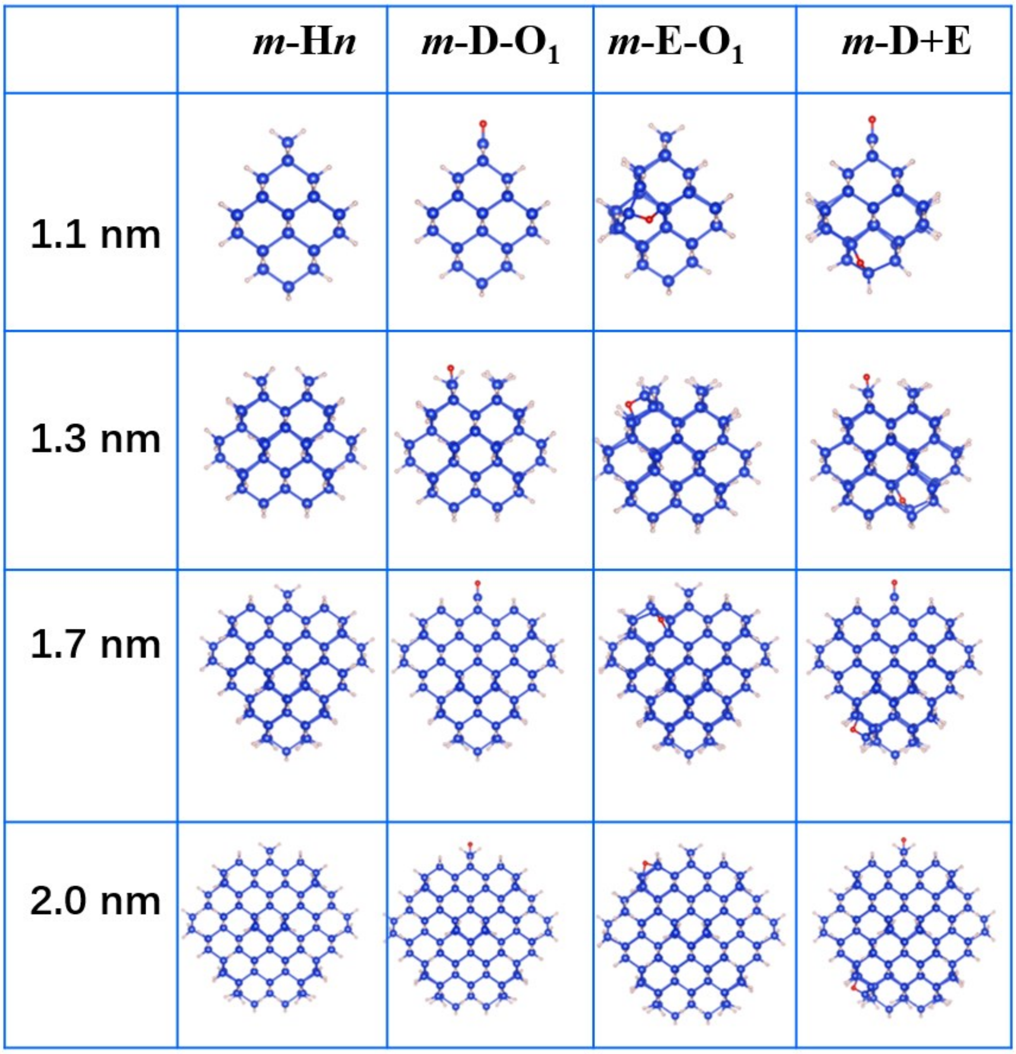

Figure S1. Structure of Si-QDs with different diameter and different passivation configurations.

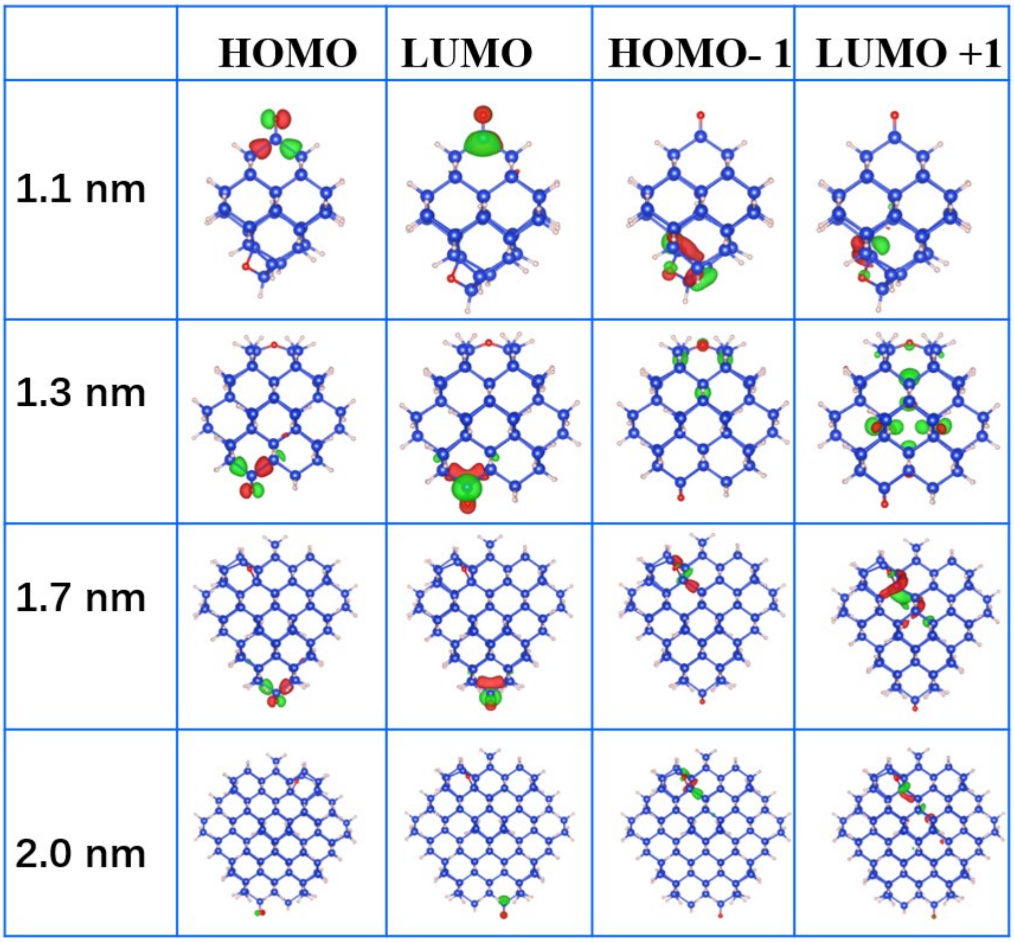

Figure S2. HOMO, LUMO, HOMO-1, and LUMO+1 real space distribution of m-D+E Si-QDs.

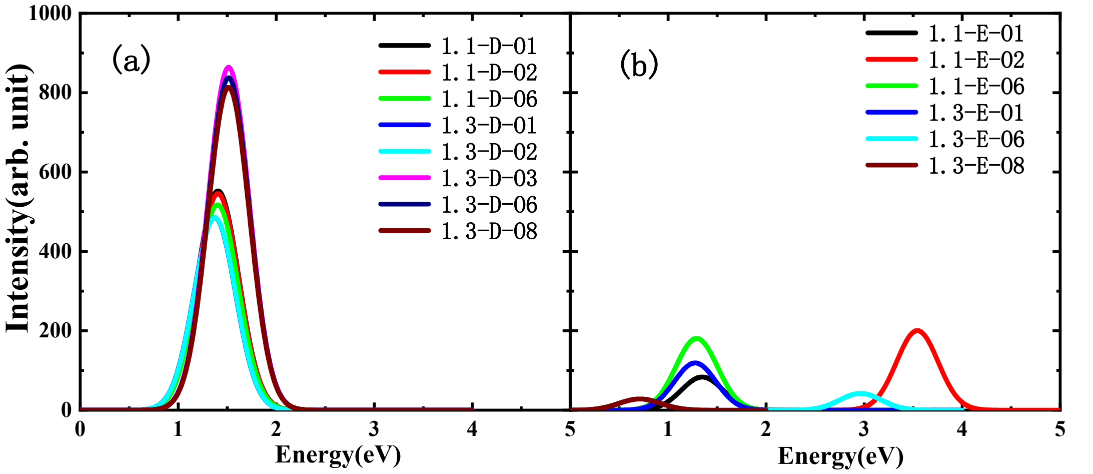

Figure S3. Emission spectrum from first singlet excited to ground state for various smaller Si-QDs with more than one oxygen atoms. (a) double bonded, (b) epoxide ring bonded

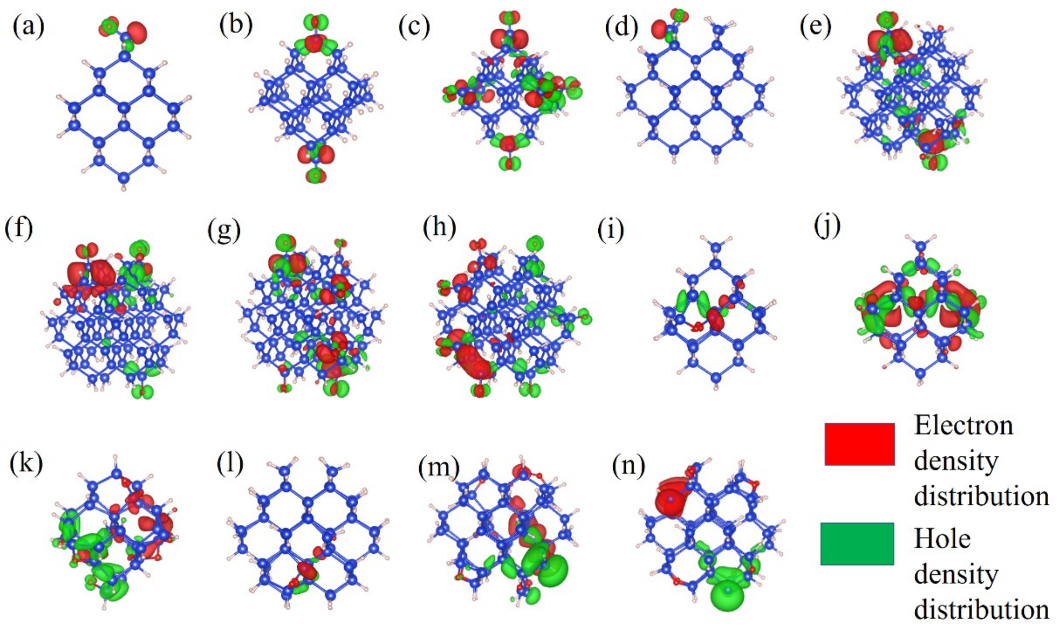

Figure S4. Electron and hole distribution of Si-QDs with more than one oxygen atoms, in which cyan regions represent electron distribution and mag regions represent hole distribution. (a) 1.1-D-O_1, (b) 1.1-D-O_2, (c) 1.1-D-O_6, (d) 1.3-D-O_1, (e) 1.3-D-O_2, (f) 1.3-D-O_3, (g) 1.3-D-O_6, (h) 1.3-D-O_8, (i) 1.1-E-O_1, (j) 1.1-E-O_2, (k)1.1-E-O_6, (l)1.3-E-O_1, (m)1.3-E-O_6, (n)1.3-E-O_8
